# Strengthening support mechanisms for performance improvement of Accredited Social Health Activists in Uttar Pradesh

**DOI:** 10.1186/1753-6561-6-S1-O8

**Published:** 2012-01-16

**Authors:** Dharmendra S Panwar, Emily Das, Vandana Naidu, Madhuri Narayanan, George Philip

**Affiliations:** 1 Project Vistaar, IntraHealth International, India

## Introduction

The National Rural Health Mission (NRHM) was launched in 2005. One of the key components of the NRHM is the identification and training of Accredited Social Health Activists (ASHA) for each village in the country. They have undergone several rounds of training and received additional training like 10-day training on child survival in Uttar Pradesh state. Despite these training programmes, significant gaps remain in their performance and their ability to translate “knowledge to practice”.

Recognising that improving performance of ASHAs is critical to achieving the outcomes of NRHM, the USAID-funded IntraHealth International led *Vistaar* project, as part of technical assistance to the Uttar Pradesh government, planned assessments to identify (1) gaps between the desired and actual performance of ASHAs, (2) key factors affecting ASHA performance, and (3) on-the-job support, capacity building and mentoring needs of ASHAs.

## Methods

### Qualitative assessments

We conducted rapid needs assessment and performance needs assessment comprising of focus group discussions and in-depth interviews with ASHAs, auxiliary nurse midwives (ANM), lady health visitors (LHV) and medical officers (MO) across five districts of Uttar Pradesh.

We used systematic random sampling to select ASHAs using the list of trained ASHAs provided by the district governments as the sampling frame. The supervisors (ANMs, LHVs and MOs) of the selected ASHAs were interviewed to assess the existing supportive supervision mechanisms for ASHAs. Content analysis of the qualitative data from in-depth interviews was carried out using N6 software package. Two performance need assessment were undertaken at two different locations with about 40 participants each, which included ASHAs, ANMs, LHVs and MOs. We attempted to understand the gaps between desired and actual performance by using root cause analysis to identify underlying factors and address the root causes at various levels.

### The intervention

The *Vistaar* Project provided technical assistance in five districts of Uttar Pradesh to regularise monthly ASHA meetings, optimise these as platforms for continued capacity building of ASHAs and enhance their interpersonal communication skills for effective transformation of knowledge to practice. The interventions were planned to reach to 10,000 ASHAs. Vistaar designed capacity building modules on supportive supervision and assisted government with training of ANMs and LHVs in five districts to provide on-site support to ASHAs during field interactions. ANMs were trained to provide problem solving /action planning support, provide positive feedback wherever possible and motivate ASHAs.

## Results

The assessment revealed that ASHAs possess basic theoretical knowledge, but significant gaps remain in application of this knowledge. ASHAs lacked operational skills to translate knowledge to practice. They also lack inter-personal communication skills required to communicate effectively and negotiate behaviour change at household level.

We also found that there is not much support or mentoring after the training programme to improve their competence. The ASHA monthly meetings remained unutilised for on-the job capacity building and supportive supervision for ASHAs. Village health and nutrition days are missed opportunities for field interaction between ASHA and ANM and providing on-site support to ASHAs.

Following the intervention, we found that where capacity building was undertaken, the ASHA meetings increased from 53% to 98%. ASHA attendance at monthly meetings improved significantly from 48% to 82%. The proportion of recently delivered women visited by ASHAs who reported use of job-aides by ASHAs increased from 10% to 22%. The proportion of recently delivered women who reported being counselled on early initiation of breastfeeding increased from 64% to 77%. The proportion of mothers visited by ASHAs at least once within 3 days of birth increased from 0.6 % to 35%.

## Discussion

Supportive supervision (including capacity building) of ASHAs can be strengthened through the existing system by making use of the monthly meetings more effectively. The meetings of ASHAs must be made regular and further opportunities to meet in smaller groups should be provided to make the meetings more effective and interactive. The results also suggest that the involvement of ANMs in meetings along with ASHAs promotes team building, provides opportunity for joint problem solving and follow-up support during field-visits.

